# Disjunct Northern Populations as Reservoirs of Evolutionary Diversity: Insights from the Aesculapian Snake (*Zamenis longissimus*)

**DOI:** 10.3390/ani15131894

**Published:** 2025-06-26

**Authors:** Ivan Rehák, Radka Musilová, Silvia Marková, David Fischer, Petr Kotlík

**Affiliations:** 1 Prague Zoo, 171 00 Prague, Czech Republic; 2 Association Zamenis, z.s., 363 01 Stráž nad Ohří, Czech Republic; zamenisos@seznam.cz; 3 Laboratory of Molecular Ecology, Institute of Animal Physiology and Genetics, Czech Academy of Sciences, 277 21 Liběchov, Czech Republic; markova@iapg.cas.cz; 4 Mining Museum Příbram, 261 01 Příbram, Czech Republic; fischer-david@seznam.cz

**Keywords:** rat snakes, isolated populations, autochthonous population, introduction, new haplotypes, *coxI*, *cytb*, genetic admixture, climate change

## Abstract

Edge populations can harbor unique genetic diversity shaped by historical isolation and play a key role in species’ resilience and range expansion under ongoing climate warming. The Aesculapian snake (*Zamenis longissimus*) reaches the northern limit of its range in Central Europe, where isolated populations may provide key insights into the species’ evolutionary potential and conservation priorities. In Bohemia (the western Czech Republic), only one reproducing population, in the vicinity of Stráž nad Ohří (SO), had previously been confirmed north of the species’ continuous distribution. Here, we report two additional reproducing populations recently discovered through long-term monitoring: one at the Želinský meander (ZM) and another in Central Bohemia (CB). The ZM population is autochthonous, viable, and genetically remarkable, harboring two divergent mitochondrial haplotypes: the widespread Eastern phylogeographical clade haplotype E1 and a novel haplotype, W10, belonging to the Western clade. This represents the first confirmed record of a Western clade haplotype in the Czech Republic, and only the second known locality within the species’ entire range where both clades coexist. In contrast, the CB population—founded by human-mediated translocation from SO—is expanding dynamically and is represented solely by E1, the only haplotype previously recorded in the country. Our study highlights the importance of incorporating genetic data into conservation planning and understanding species’ evolutionary potential. The mitochondrial diversity uncovered at the ZM exemplifies how historical processes, isolation, and lineage mixing shape contemporary genetic structure. Preserving such populations, which retain unique evolutionary diversity, will be critical for maintaining the resilience of *Z. longissimus* in Central Europe. More broadly, disjunct northern populations may serve as reservoirs of genetic diversity, enhancing adaptive potential and supporting future range expansion under climate change. Recognizing and conserving this diversity is essential not only for local persistence but also for species-level resilience in a rapidly changing environment.

## Simple Summary

Isolated populations at the edges of a species’ range can play an outsized role in helping species survive environmental change. The Aesculapian snake, a rare and protected species in the Czech Republic, reaches the northern edge of its main continuous range in Bohemia, the western part of the country. We characterized two newly discovered, isolated, and viable populations: one, a natural relict surviving in a secluded river meander, preserves two distinct genetic lineages—an extremely rare and valuable finding. The other, an expanding population in the river valleys of Central Bohemia, began as a human introduction but has since grown rapidly, thriving in both natural landscapes and human-altered environments. Our findings show that even isolated populations can hold critical genetic diversity and could help the species adapt and shift its range northward as the climate warms. Protecting this diversity now is vital for ensuring the long-term survival and resilience of the Aesculapian snake in a fast-changing world.

## 1. Introduction

Edge populations are often overlooked in conservation planning, yet they can preserve unique genetic diversity shaped by historical isolation and may play a disproportionate role in species’ resilience and range shifts under environmental change. Understanding the origins, genetic composition, and viability of such populations is critical for informing conservation strategies, particularly under ongoing global climate change [1]. Genetic assessments help distinguish natural from anthropogenic populations and are vital for prioritizing conservation actions and guiding management interventions, such as translocations or assisted gene flow [2].

The Aesculapian snake, *Zamenis longissimus* (Laurenti, 1768), one of Europe’s largest snakes, holds particular cultural significance due to its ancient symbolic association with healing, apothecary practices, and medicine, as well as its role in mythology [3,4]. As part of the relatively rapid evolution of the rat snakes of the genus *Zamenis* in the late Neogene, it was *Z. longissimus* and its sister *Z. lineatus* (Camerano, 1891) that split off as the last, youngest species during the phylogeny of the genus [5]. Among the six currently recognized species of West Palearctic rat snakes in the genus *Zamenis* Wagler, 1830 [5,6], *Z. longissimus* has the broadest range, spanning from northeastern Spain to the Black Sea (across southern and central France, the northern and central Apennine Peninsula, southern Switzerland, Austria, Slovakia, Hungary, and the Balkan Peninsula).

**Figure 1:**
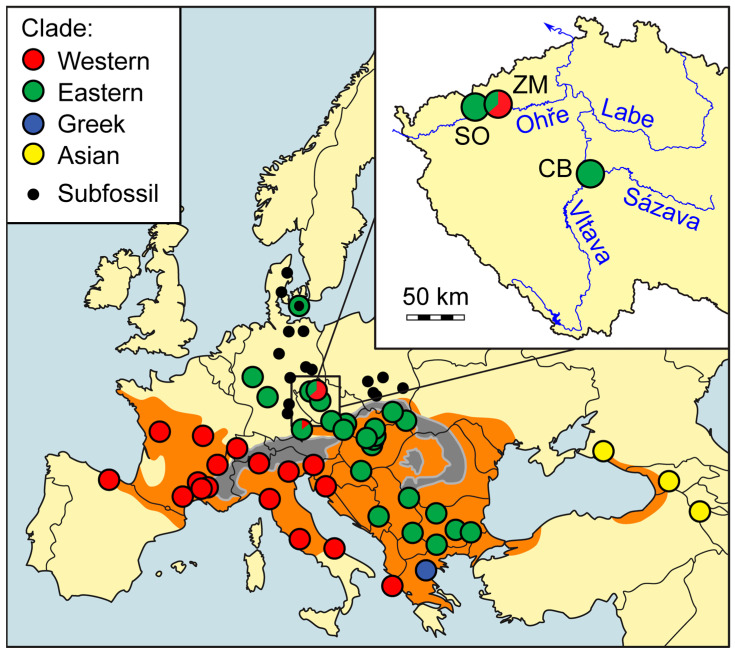
Geographic distribution and phylogeographic structure of *Zamenis longissimus*, showing evidence of the coexistence of two clades in the Czech Republic (new data from this study). The current distribution range (shaded in orange) and Holocene subfossil records from regions north of this range (black circles), including a record from Denmark genetically assigned to the Eastern clade [16], are based on previously published data. Major mountain ranges, including the Alps and Carpathians, are indicated in grey. The inset map details clade occurrence at the three study sites within Bohemia (the western part of the Czech Republic), with both Western and Eastern clades found at the Želinský meander (ZM). In contrast, individuals from Stráž na Ohří (SO) and Central Bohemia (CB) belong exclusively to the Eastern clade. Overview map adapted from Ref. [7]; basemap of Bohemia adapted from Caroig—Own work, CC BY-SA 2.5, https://commons.wikimedia.org/wiki/File:Central_Bohemian_Region_(CZE)_-_location_map.svg (accessed on 10 April 2025).

Phylogeographically, four well-defined groups can be distinguished within the species [7] (Figure 1). From the perspective of the current European distribution, the major ones are (i) the Western clade, inhabiting the western part of the range west and south of the Alps and along the Adriatic Sea, and (ii) the Eastern clade, occurring east and northeast of the Alps and on the Balkan Peninsula (excluding the Adriatic coast and Greece), which spread from the Balkan glacial refugium to central and northern Europe, reaching as far as Denmark during the Holocene Climatic Optimum. The remaining two clades have a much smaller geographical distribution. The Asian clade (iii) is native to the eastern Black Sea area, and the last one (iv) is from Greece. The distribution of *Z. longissimus* is fragmented: while relatively continuous across southern and central Europe, the species reaches the northern limit of its range near the 50th parallel, beyond which only isolated populations persist. For more detailed information on the range, see Refs. [8,9,10,11,12].

These northern disjunct populations are likely relicts of a broader Holocene distribution, when climatic conditions allowed the species to expand further north (Figure 1), as evidenced by subfossil records from Germany, Poland, and Denmark [10,13,14,15,16]. Some isolated populations, such as those in the United Kingdom, are the result of documented human-mediated introductions [9,17,18,19], while others represent ancient, natural relicts. The existence of these populations is often closely linked to anthropogenic factors and is now frequently supported by active conservation management [10,11,20,21]. Although *Z. longissimus* is classified as Least Concern by the global IUCN [12], it is listed under Annex IV of the EU Habitats Directive, which requires strict protection. A European action plan has also been established for its conservation [9]. Within the Czech Republic, it is legally protected and classified as Critically Endangered. In 2008, an action plan for *Z. longissimus* was established by the Nature Conservation Agency of the Czech Republic.

In the Czech Republic, populations in Moravia (Podyjí and White Carpathians) are connected to the species’ continuous range, but Bohemian populations are geographically isolated [10,11] (Figure 1). Until recently, only one reproducing population had been confirmed in Bohemia (the western part of the Czech Republic), near Stráž nad Ohří (SO) along the Ohře River, over 200 km from the continuous range [10,11,22,23]. Long-term monitoring has revealed that this population is existentially threatened.

The recent discovery of two additional reproducing populations in Bohemia is therefore of major conservation importance. One population, located at the Želinský meander (ZM) of the Ohře River, approximately 20 km downstream from SO, appears to be a naturally surviving relict population and is thus particularly significant (own data). The other, in Central Bohemia (CB) in the middle Vltava–lower Sázava river regions, likely originated from snakes deliberately introduced in the 1980s, sourced from SO, according to personal communication with the individual responsible for the introduction, as conveyed directly to the first author.

Assessing the genetic structure of these populations is essential for elucidating their evolutionary origins, evaluating their conservation significance, and guiding future management strategies [1]. Populations at range margins are often small and fragmented, making them susceptible to genetic drift and inbreeding—processes that can reduce genetic diversity and compromise adaptive potential. However, if such isolated populations retain unique genetic lineages or exhibit signatures of past lineage mixing, they may play a disproportionately important role in shaping the species’ adaptive capacity and range dynamics [24].

In this study, we investigate the distribution and genetic composition of the two newly discovered isolated Bohemian populations (ZM and CB) of *Z. longissimus*, based on mitochondrial DNA sequences, and compare them with the previously known SO population. Our findings extend the documented northern range of the species, elucidate patterns of mitochondrial diversity, and highlight the importance of disjunct populations as reservoirs of evolutionary potential and as critical targets for conservation efforts in a rapidly changing climate.

## 2. Materials and Methods

### 2.1. Study Areas

#### 2.1.1. Želinský Meander

**Figure 2:**
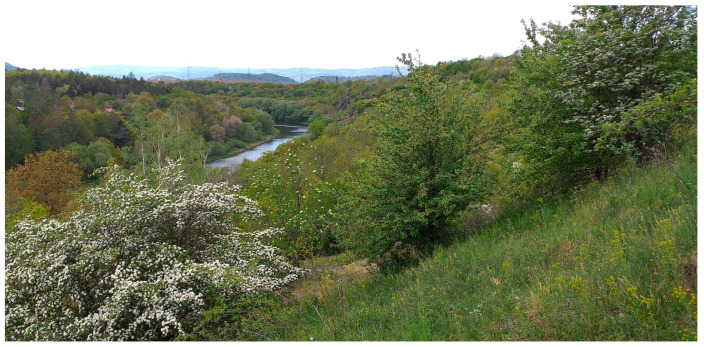
Želinský meander (ZM) of the Ohře River (6 May 2020).

The ZM is a protected natural monument and a site of European importance, located in the canyon-like valley of the Ohře River near Kadaň (approximate coordinates: 50°22′ N, 13°19′ E) (Figure 2). The protected area covers approximately 185 ha, with elevations ranging from 270 to 331 m above sea level. It hosts a remarkable geomorphological and biological diversity, including habitats such as muddy riverbanks, European dry heaths, and continental deciduous scrub, supporting numerous endangered and protected plant and animal species [25].

The site lies in a moderately warm climatic region, characterized by long, warm, dry summers and short, moderately warm, very dry winters with brief snow cover. Annual precipitation ranges between 550 and 650 mm (350–400 mm during the growing season), and the number of summer days is 40–50, with an average July temperature of 17–18 °C [26,27].

#### 2.1.2. Central Bohemia

**Figure 3:**
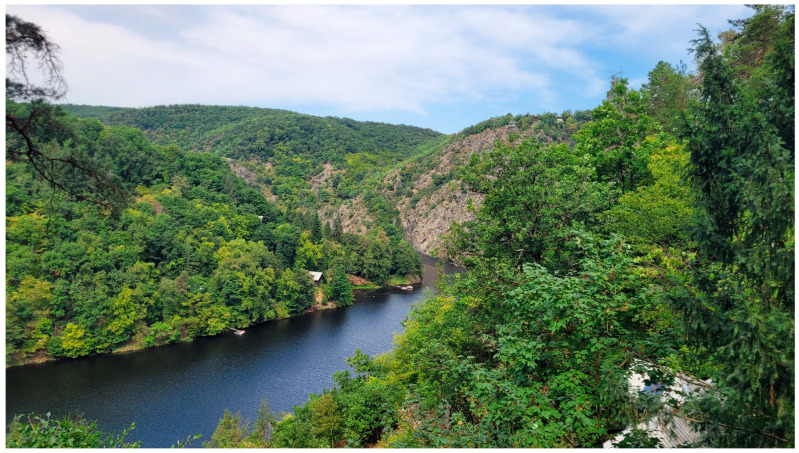
The valley of the Vltava River near the village of Krňany in Central Bohemia (CB) (25 August 2023).

The CB study area spans the Vltava and Sázava river canyons and their surrounding landscapes (approximate coordinates: 49°50′ N, 14°28′ E), combining relatively undisturbed habitats with a mosaic of cultural landscapes shaped by human activity. Elevations range from 200 to 448 m above sea level. The slopes of the river valleys are predominantly covered by deciduous forests, interspersed with rocky steppes on south-facing exposures and deeply incised stream valleys (Figure 3).

A large part of the local Vltava canyon is influenced by the existence of the dam and the connecting Štěchovice reservoir. The area includes protected sites such as the Kobylí Draha Nature Reserve and the Medník National Nature Monument, as well as extensive recreational zones with cottage settlements. Like the ZM, CB is located in a moderately warm climatic region and shares similar precipitation, temperature, and seasonal patterns [26,27]; the regional values provided for the ZM apply to this site as well.

### 2.2. Monitoring and Survey Data

#### 2.2.1. Želinský Meander

Herpetological surveys in the ZM have been ongoing for over two decades, initially focusing on *Natrix tessellata* in 2009–2010. Intensive systematic monitoring specifically targeting *Z. longissimus* was conducted from 2020 to 2024, with 53 visits distributed over five years (2020: 8 visits, 2021: 10, 2022: 12, 2023: 13, 2024: 10).

Monitoring followed a standardized 2 km transect route during each visit. Six fixed sheets of firm foil were installed along the route to serve as artificial shelters for reptiles. Any Aesculapian snake encountered was photographed in detail and/or marked with scale clips to facilitate individual identification in case of recapture.

#### 2.2.2. Central Bohemia

Monitoring in CB was conducted from 2021 to 2024, totaling 53 visits. Unlike the structured transect-based approach at the ZM, the Central Bohemian survey involved exploratory monitoring aimed at mapping the current distribution of *Z. longissimus* within and beyond the presumed core area. Surveys targeted both confirmed sites and adjacent potentially suitable habitats to delineate the species’ range in the middle Povltaví and lower Posázaví regions.

### 2.3. Genetic Analysis

#### 2.3.1. Samples

In total, 37 *Z. longissimus* samples were used, including 10 from SO, 11 from the ZM, and 16 from CB. Samples were obtained during prior field monitoring through non-invasive or minimally invasive methods. Ventral scale clips collected during individual marking and preserved in ethanol served as the primary genetic samples (8 from SO, 6 from the ZM, and 8 from CB). Additionally, oral swabs were taken from 4 individuals at the ZM and also preserved in ethanol. Muscle tissue was sampled from two road-killed individuals (1 from the ZM and 1 from CB). Shed skins were collected opportunistically when found in the field (2 from SO and 7 from CB).

All procedures were carried out in accordance with ethical standards to minimize animal stress and in compliance with Czech legislation (permit numbers: KK/1098/ZZ/20-4, KUUK/057806/2021, and 099683/2024/KUSK). The samples used in this study were obtained during routine monitoring activities conducted as part of the national biodiversity monitoring program coordinated by the Nature Conservation Agency of the Czech Republic.

#### 2.3.2. DNA Extraction and Sequencing

Genomic DNA was extracted from all sample types using the DNeasy Blood & Tissue Kit (Qiagen, Hilden, Germany), following the manufacturer’s protocol. Two mitochondrial gene segments—cytochrome c oxidase subunit I (*coxI*) and cytochrome b (*cytb*)—were targeted for analysis. These markers were selected due to their high utility in reptile phylogeography, their proven resolution at the intraspecific level, and their widespread use in previous studies of *Z. longissimus* and related species [7,16], enabling direct comparison with existing data. PCR amplification was carried out using primers and protocols previously validated for *Z. longissimus* [7,16]. The resulting amplicons were purified and sequenced in both directions using Sanger sequencing, performed by a commercial provider (Macrogen, Amsterdam, The Netherlands). Sequences were assembled in CodonCode Aligner v. 11.0.1 (CodonCode Corporation). To verify their identity, sequences were compared against the NCBI nucleotide database using BLAST+ v. 2.16.0 (NCBI, Bethesda, MD, USA). The sequences of *coxI* and *cytb* were then concatenated to produce a 1638 bp alignment for downstream analyses.

To place the newly obtained sequences in a broader phylogeographic context, we incorporated 28 unique mitochondrial haplotypes from *Z. longissimus* described by Musilová et al. [7], representing the Eastern, Western, Asian, and Greek clades of the species. One haplotype each of *Z. lineatus* and *Z. persicus* was also included to provide outgroup comparisons [7]. Additionally, we incorporated two haplotypes identified by Allentoft et al. [16] from museum specimens of *Z. longissimus* originating from an extinct population in Denmark: HK2489, which is identical to the Eastern clade haplotype E1 [7], and R8974, a distinct haplotype within the same clade.

#### 2.3.3. Data Analysis

Evolutionary analyses were conducted using MEGA X v. 10.1.7 [28]. Model selection was performed separately for each gene and for the concatenated dataset, using both the Bayesian Information Criterion (BIC) and the corrected Akaike Information Criterion (AICc). The TN93+G model (Tamura–Nei with gamma-distributed rate variation among sites) was identified as the best fit for all datasets under at least one criterion. Therefore, a maximum likelihood phylogenetic tree was reconstructed using this model.

In addition to tree-based phylogenetic analysis, relationships among haplotypes were visualized using a median-joining network constructed in NETWORK v. 10.2.0.0 (Fluxus Technology Ltd., Colchester, UK).

Unique haplotypes identified among the newly sequenced individuals were compared with previously published data to determine their correspondence to known haplotypes or, if unmatched, to confirm them as novel variants. These comparisons were used to evaluate the genetic distinctiveness and phylogeographic affinities of the newly discovered Czech populations of *Z. longissimus*.

## 3. Results

### 3.1. Želinský Meander

**Figure 4:**
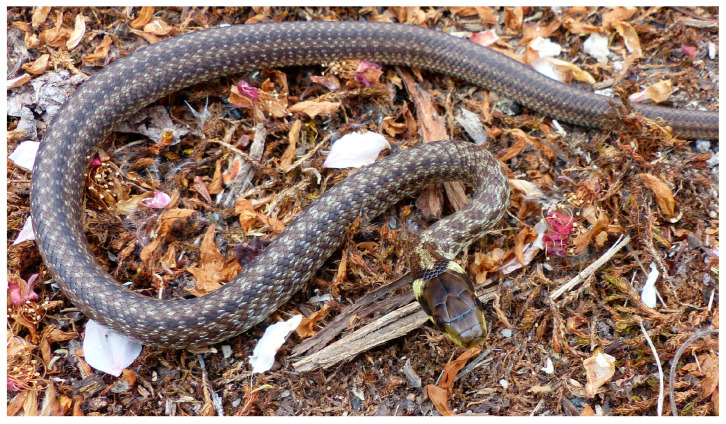
A juvenile Aesculapian snake (*Zamenis longissimus*) documented on 1 May 2020 at the Želinský meander (ZM), providing evidence of local reproduction.

Despite intensive field surveys beginning in 2009–2010, the occurrence of *Z. longissimus* in the ZM was documented for the first time only in 2017 (adult observed on 5 June 2017). However, a major breakthrough occurred in April 2020, when three adult females and one juvenile (from the previous year’s cohort; Figure 4) were recorded, indicating the presence of a reproducing local population. This prompted intensive targeted monitoring of *Z. longissimus* from 2020 to 2024.

During 53 survey visits between 2020 and 2024, a total of 80 sightings of *Z. longissimus* were recorded: 33 males, 22 females, 5 unsexed adults, 4 subadults, and 16 juveniles (after their first overwintering), with at least eight confirmed recaptures. The occupied area was approximately 2 km^2^.

*Zamenis longissimus* was found syntopically with *Natrix natrix*, *N. tessellata*, *Coronella austriaca*, *Lacerta viridis*, and *Anguis fragilis*. Compared to the nearby SO population, snakes at the ZM appeared less dependent on anthropogenic structures and were more frequently encountered in natural habitats.

**Figure 5:**
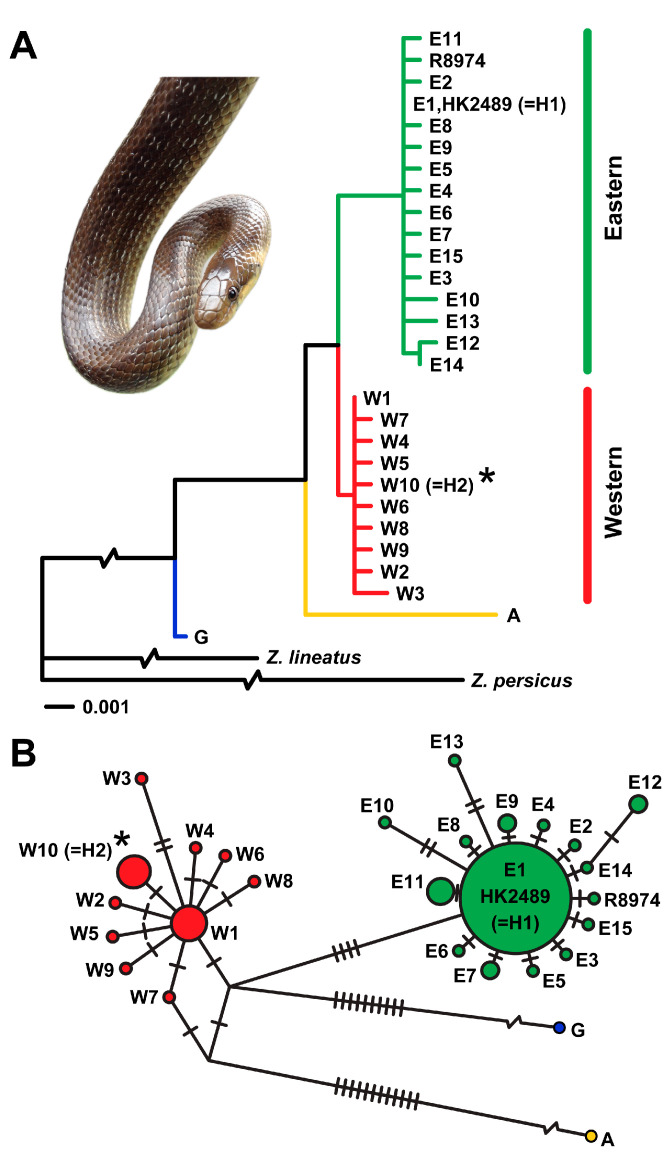
Phylogenetic evidence for a novel haplotype and the presence of two clades of *Zamenis longissimus* in the Czech Republic. (**A**) Maximum likelihood phylogeny of haplotypes. Haplotypes H1 and H2 were detected in the Czech Republic, with H1 found at all three studied sites—Stráž nad Ohří (SO), Želinský meander (ZM), and Central Bohemia (CB)—and corresponding to the common Eastern clade haplotype E1. H2, designated as W10 following the nomenclature established by Musilová et al. [7] and marked with an asterisk, was identified for the first time and found exclusively at ZM. The tree includes additional haplotypes from previous studies [7,16], including sequences from the extinct Danish population (HK2489 and R8974), to provide phylogenetic context. For clarity, three long branches have been shortened to one-tenth of their actual length. An inset photograph shows a *Z. longissimus* individual from SO. (**B**) Unrooted median-joining network of the same haplotypes, showing the inferred number of mutational steps (indicated by crossbars) and relative haplotype frequencies (reflected in circle sizes). For clarity, two long connections were visually shortened.

Mitochondrial DNA analyses revealed two distinct haplotypes among the 11 sequenced individuals from the ZM. Four individuals carried haplotype H1, corresponding to the widely distributed Eastern clade haplotype E1 (Figure 5), previously reported, for example, from SO (Czech Republic), Schlangenbad (Germany), and Denmark [7,16]. The remaining seven individuals displayed a novel haplotype H2. Phylogenetic and network analyses placed haplotype H2 within the Western lineage of *Z. longissimus*, differing from the basal Western haplotype W1 by a single nucleotide substitution in the *cytb* gene (Figure 5). Therefore, following the haplotype nomenclature established by Musilová et al. [7], haplotype H2 was designated as W10. In contrast, all 10 individuals sequenced from SO carried only the Eastern haplotype H1 (= E1).

### 3.2. Central Bohemia

The occurrence of *Z. longissimus* in CB was first documented in 2016 (adult observed on 16 June 2016). Systematic monitoring of the middle Vltava–lower Sázava region in 2020–2024 yielded observations of 142 *Z. longissimus* individuals: 7 males, 6 females, 109 unsexed adults, 6 subadults, and 14 juveniles. Additionally, 58 developing eggs were recorded, further confirming successful reproduction. The currently known distribution area covers approximately 20 km^2^, with the direct distance between the most distant confirmed occurrences measuring 7.8 km. Approximate coordinates of extreme points of confirmed occurrence are 49°53′ N, 14°23′ E; 49°52′ N, 14°29′ E; 49°50′ N, 14°28′ E; 49°51′ N, 14°27′ E; and 49°51′ N, 14°24′ E. Elevations of documented localities range from 203 to 417 m above sea level. 

Most observations were concentrated near human settlements (gardens, farms, and abandoned buildings, municipal waste dumps), suggesting a strong association with anthropogenic habitats, particularly for reproduction and hibernation. However, some individuals were also documented in more natural habitats farther from settlements.

Genetic analysis of 16 individuals from CB revealed the exclusive presence of haplotype H1, corresponding to the Eastern clade haplotype E1 [7] (Figure 5). No additional haplotypes or evidence of mitochondrial admixture were detected. These results indicate matrilineal genetic uniformity within the CB population, consistent with exclusive affiliation to the Eastern lineage of *Z. longissimus*.

## 4. Discussion

### 4.1. Želinský Meander

Occasional records of the Aesculapian snake along the Ohře River below Kadaň date back to the 1930s, where the species was already described as rare. Since the late 1990s, only isolated and irregular observations have been made, despite intensive targeted surveys [11,23,29]. The management plan for the Želinský Meander Nature Reserve for 2013–2022 [25] does not mention *Z. longissimus* at all. The discovery of a well-established, reproducing population in the ZM is therefore highly significant—historically, for current conservation practice, and for understanding the species’ biogeography and genetic diversity.

Our genetic analyses reveal that the ZM population is exceptional both within the Czech Republic and in the species’ overall phylogeographic framework. Until now, all Czech individuals analyzed shared a single mitochondrial haplotype (E1), characteristic of the Eastern clade. In contrast, the ZM harbors both this common haplotype and a novel haplotype (W10) belonging to the Western clade—representing the first confirmed record of a Western haplotype in the Czech Republic, and only the second known population across the species’ entire range where Eastern and Western clades coexist (after Burghausen in southern Bavaria; [7]; Figure 1). This finding identifies the ZM as an unexpected hotspot of mitochondrial diversity within an ecologically isolated population.

The presence of divergent haplotypes at the ZM suggests two potential scenarios regarding the origin of its genetic diversity. One plausible explanation is natural secondary contact between historically isolated lineages, likely linked to postglacial range dynamics. Although the contemporary distributions of the Eastern and Western clades do not overlap north of the Alps, *Z. longissimus* extended as far north as Denmark during the mid-Holocene climatic optimum [7,16]. Analyses of museum specimens from Denmark (dating back to the early 19th century) confirm Eastern clade haplotypes [16], and all extant relict populations studied to date in the Czech Republic and Germany also carry Eastern haplotypes [7]. However, a unique coexistence of both clades at the ZM suggests that a contact zone may have once existed in regions north of the Alps, where the species has since largely disappeared. The persistence of both matrilines at the ZM could therefore result from long-term survival in isolated microhabitats, potentially followed by recent local expansion or habitat reconnection. This would help explain how two divergent matrilines may have persisted over time despite genetic drift, particularly in a small or fluctuating population.

An alternative explanation could be a human-mediated introduction of individuals from a Western European source population. However, the absence of the widespread haplotype W1—commonly found in Western populations—and the presence of a unique haplotype W10 at the ZM argue against a recent introduction. Coupled with the lack of historical records or practical evidence supporting translocation, we consider this scenario unlikely. Instead, we interpret the ZM population as an autochthonous relict population, reflecting historical natural processes.

Lineage coexistence at the ZM contrasts with patterns in other reptiles with broader historical ranges and northern relict populations. For example, the European pond turtle (*Emys orbicularis*) shows low mitochondrial diversity in northern populations without co-occurring lineages [30]. This difference underscores the need to consider species-specific evolutionary histories and genetic structures of relict populations.

Comparison with the nearby SO population, located just 20 km upstream, further underscores the distinctiveness of the ZM. The SO population, despite extensive study ([7,16], this study), appears to contain only the Eastern clade haplotype. This suggests either a lack of historical gene flow between SO and the ZM or unidirectional gene flow downstream. The presence of both Eastern and Western lineages exclusively at the ZM highlights how fine-scale genetic structure can exist even over short geographic distances.

Such genetic differentiation carries significant conservation implications. Population reinforcement strategies often assume genetic homogeneity within national or regional contexts, but our findings caution against this assumption. Important differences in genetic diversity and lineage structure may exist among relict populations both within species and between species, highlighting the complexity of genetic variation. Populations that seem similar may have different evolutionary histories and genetic composition. Translocating individuals without considering these differences can risk the loss of important genetic variation [1].

The discovery of divergent matrilines at the ZM raises the possibility of broader genomic admixture. Populations combining lineages shaped in different glacial refugia could potentially have enhanced adaptive potential under climate change [24]. Conversely, small, isolated populations with limited gene flow may have reduced genetic diversity, potentially increasing their vulnerability. Our findings suggest that even isolated relict populations may represent reservoirs of substantial evolutionary diversity and could serve as important sources for assisted gene flow to support genetically depleted populations [2]. Effective conservation interventions should therefore be guided by a nuanced understanding of existing genetic variation, its origins, and its evolutionary significance [1].

We acknowledge that our study is limited by its focus on mitochondrial DNA and modest sample sizes. Nevertheless, the key finding—the coexistence of two phylogeographic lineages within a small, isolated population—is robustly supported by the data. While expanded sampling might reveal additional genetic diversity or lineage overlap, this remains the first documented case in a northern edge population and only the second across the species’ range despite over 100 *Z. longissimus* having been sequenced ([7,16], this study).

The ZM population thus exemplifies the importance of integrating genetic data into conservation decision-making. Its mitochondrial diversity not only highlights an overlooked conservation priority but also provides a compelling case study of how range dynamics, local isolation, and lineage mixing can shape contemporary genetic structure. Preserving the full spectrum of this variation—including rare or locally unique variants—will be essential for maintaining the evolutionary resilience of *Z. longissimus* in Central Europe, particularly under accelerating environmental change.

More broadly, isolated populations at the northern margin of a species’ range may play an important role in future range expansion under ongoing climate warming. Although our study is based solely on mitochondrial data, phylogeographically distinct populations, such as those shaped in different glacial refugia, may retain unique genetic signatures that contribute to overall evolutionary potential [24]. In this context, the distinctiveness of northern disjunct populations may reflect not only historical biogeography but also underscore their potential relevance in future conservation planning [1]. Recognizing and protecting such populations is important, not only for local persistence but also for maintaining phylogeographic and evolutionary diversity that may support species-level resilience in a changing climate.

### 4.2. Central Bohemia

The CB population of *Z. longissimus* has a relatively recent history. According to information provided directly to the first author by the individual involved, snakes from the SO population were deliberately introduced to Kobylí Draha Nature Reserve, approximately 115 km away, in the early 1980s as a rescue effort from areas within SO threatened by habitat destruction. Subsequent photographic records (e.g., May 1993 on iNaturalist) and later observations confirmed the species’ presence in the region [31]. Our field surveys have since demonstrated that the CB population is successfully reproducing and expanding both in numbers and spatial extent.

The current range of the CB population covers approximately 20 km^2^; however, the species likely inhabits a larger area throughout the middle Vltava–lower Sázava river regions. The most distant confirmed records from the introduction site (Kobylí Draha NR) extend up to 4.6 km northwest (near Davle), 3.4 km northeast (Rakousy), and 3 km south-southeast (Teletín). Given the habitat potential of the landscape, further expansion is likely.

The CB population represents an important reservoir of *Z. longissimus* in Central Europe. Isolated by approximately 155 km from the nearest populations connected to the continuous range in Podyjí (southern Moravia), and by 115 km from the SO and ZM populations, the CB population occupies a strategically significant position. Recent ecological niche models developed to assess the potential distribution of *Z. longissimus* project a northward expansion of suitable habitat for *Z. longissimus* in Europe under various future climate scenarios, with gains in northern areas potentially offsetting losses at southern range edges [32]. These projections underscore the potential future importance of northern-edge populations in facilitating range shifts, especially in light of the documented warming trends for the Czech Republic [33]. The extent to which this potential is realized will depend on local landscape features, including habitat connectivity and the availability of biocorridors.

Genetic analyses revealed that all individuals sampled from CB carried the Eastern mitochondrial haplotype E1, identical to the single haplotype found in the SO population [7], consistent with the reported introduction from SO to CB. While mitochondrial data alone cannot entirely rule out natural colonization or introduction from elsewhere within the range of the Eastern clade, the existence of a deliberate introduction from SO approximately forty years ago, combined with genetic evidence matching the SO haplotype, strongly supports an anthropogenic origin for the CB population.

These findings emphasize the importance of combining genetic analyses with contextual historical information, including personal accounts, when investigating the origins and conservation significance of isolated populations. While mitochondrial DNA offers limited resolution for assessing post-introduction genetic variation, it is invaluable for reconstructing historical scenarios, especially when paired with documented translocation events.

## 5. Conclusions

Together, our findings highlight the conservation importance of isolated *Z. longissimus* populations at the northern edge of the species’ range. The ZM and CB populations illustrate two distinct but complementary conservation challenges and opportunities. The ZM population, as a natural relict harboring genetic diversity from both Eastern and Western clades, represents an irreplaceable component of the species’ evolutionary legacy and underscores the importance of preserving naturally occurring genetic variation. In contrast, the CB population, although of anthropogenic origin, demonstrates the potential for isolated populations to establish, expand, and contribute to future range shifts under climate change. Both natural and human-facilitated populations may thus have valuable roles in conservation—provided that management actions are guided by a detailed understanding of each population’s history, genetic structure, and ecological potential.

To further refine conservation strategies and fully assess genetic resilience, future studies incorporating nuclear genetic markers will provide valuable complementary insights. Recognizing and conserving the genetic and ecological distinctiveness of range-edge populations is essential not only for local population survival but for ensuring species-wide adaptability in a rapidly changing environment.

Our findings have relevance for the ongoing update of the *Z. longissimus* action plan under the umbrella of the Nature Conservation Agency of the Czech Republic. In particular, we suggest that reports of undocumented populations be prioritized for verification, as they may harbor unrecognized genetic diversity with conservation significance. Given the genetic distinctiveness and isolation of some populations identified in this study, caution is warranted when considering any future translocation or “genetic refreshment” efforts. The transfer of individuals between distinct populations should be carefully evaluated to avoid unintended disruption of local genetic structure. Further research will be essential to formulate appropriate, context-specific conservation strategies. The integration of genetic data into conservation planning will benefit from continued collaboration among researchers, practitioners, and policy makers.

## Data Availability

Genetic data are available in a publicly accessible repository in GenBank (accession numbers PV780469–PV780470 and PV791207–PV791208).

## Abbreviations

The following abbreviations are used in this manuscript:

IUCN	International Union for Conservation of Nature
SO	Municipality of Stráž nad Ohří and its surroundings
ZM	Želinský meander Nature Monument
CB	The middle Povltaví and lower Posázáví in Central Bohemia
